# Intrauterine administration of peripheral mononuclear cells in recurrent implantation failure: a systematic review and meta-analysis

**DOI:** 10.1038/s41598-019-40521-w

**Published:** 2019-03-07

**Authors:** Kayhan Yakin, Ozgur Oktem, Bulent Urman

**Affiliations:** Department of Obstetrics and Gynecology, Koc University School of Medicine, Istanbul, Turkey

## Abstract

It has been proposed that intrauterine administration of peripheral blood mononuclear cells (PBMCs) modulates maternal immune response through a cascade of cytokines, chemokines and growth factors to favor implantation. We conducted a meta-analysis to verify the effect of intrauterine PBMC administration on the outcome of embryo transfer in women with recurrent implantation failure (RIF). All relevant trials published in PubMed, Web of Science and Cochrane library databases were searched. Two randomized controlled trials and three cohort studies (1173 patients in total) matched the inclusion criteria. No differences in live birth rates were seen between the PBMC-treated patients and controls (OR: 1.65, 95% CI: 0.84–3.25; p = 0.14; *I*^2^: 66.3%). The clinical pregnancy rate was significantly higher in women who received intrauterine PBMCs before embryo transfer compared with those who did not (OR: 1.65, 95% CI: 1.30–2.10; p = 0.001, heterogeneity; *I*^2^: 60.6%). Subgroup analyses revealed a significant increase in clinical pregnancy rates with the administration of PBMCs in women with ≥3 previous failures compared with controls (OR: 2.69, 95% CI: 1.53–4.72; p = 0.001, *I*^2^: 38.3%). In summary, the data did not demonstrate an association between the administration of PBMCs into the uterine cavity before fresh or frozen-thawed embryo transfer and live birth rates in women with RIF. Whether intrauterine PBMC administration significantly changes live birth and miscarriage rates requires further investigation.

## Introduction

Procedures or medications with limited or unproven effectiveness are typically offered to couples with repeated implantation failure who desperately seek novel therapies. Implantation failure has been related to a myriad of problems ranging from anatomical uterine defects to more complex etiologies, such as suboptimal embryo quality or decreased endometrial receptivity; however, the etiology remains unexplained in the majority of cases^1^.

Despite the lack of a uniform definition, recurrent implantation failure (RIF) is generally defined as the absence of a clinical pregnancy after two consecutive fresh or frozen embryo transfer cycles involving the transfer of at least four cleavage stage embryos or two good quality blastocysts. The current treatment of RIF is largely empirical and based on poor scientific evidence. There is a sizable amount of literature relating to the management options in RIF cases targeting either the embryo (assisted hatching, aneuploidy screening), the endometrium (endometrial co-culture, scratching, receptivity assays, short-term copper intrauterine device insertion, freezing-all strategy), the maternal immune system (steroids, aspirin, heparin, immunoglobulins, anti-TNF antibodies, granulocyte colony-stimulating factor, intralipid infusions), or sperm (higher magnification sperm selection, microchips), but none of these techniques been proven beyond reasonable doubt to have a therapeutic effect^2,3^.

During the last decade, particular interest has focused on the role of endocrine-immune interactions in the implantation process. The interplay between the invading embryo and mononuclear cells that come into contact with trophoblasts of embryonic origin through a cascade of cytokines, chemokines and growth factors actively occurs at the implantation site. Based on this rationale, it has been proposed that modulating the maternal immune response to the invading embryo might improve the clinical outcome in women who fail to achieve pregnancy despite the transfer of good quality embryos. In 2006, researchers reported that women with RIF who received intrauterine peripheral blood mononuclear cells (PBMCs) cultured with human chorionic gonadotropin (hCG) experienced increases in implantation rates^4^. PBMCs are defined as any blood cell with a round nucleus, such as B and T lymphocytes, monocytes or macrophages^5^. Since then, a few controlled studies, including two randomized controlled trials with substantial differences in methodologies and small sample sizes, reported promising findings with the use of PBMCs in women with RIF^6–9^.

The primary objective of this systematic review and meta-analysis was to assess the available evidence on the intrauterine administration of PBMCs in women with implantation failures, provide an estimate of the average treatment effect, and recommend how to improve the design of future trials.

## Results

### Search results, study characteristics, and quality assessment

**Figure 1:**
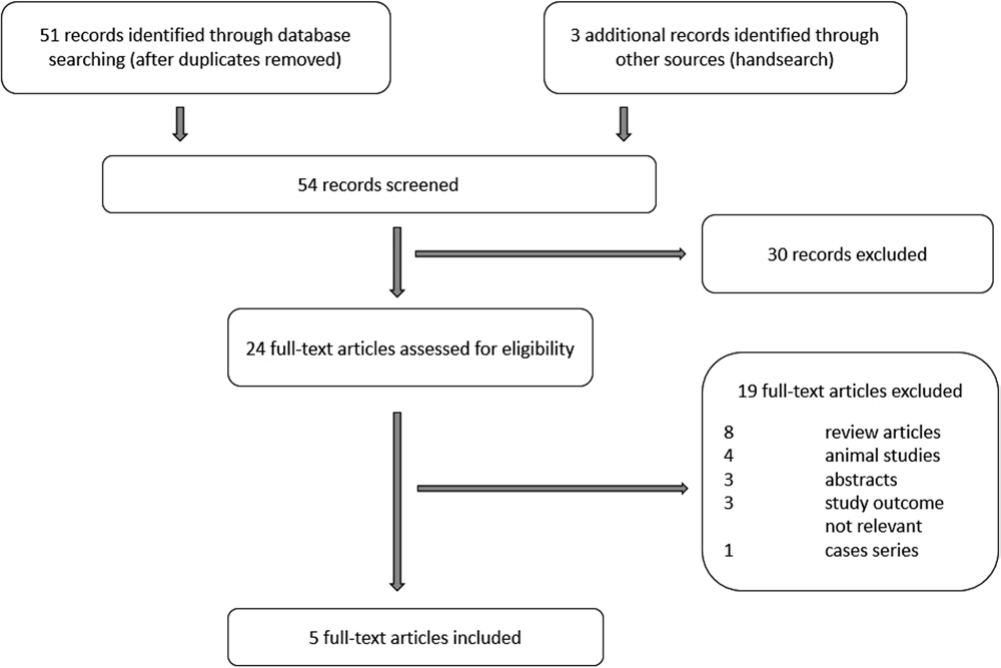
PRISMA flow diagram for study selection.

The Preferred Reporting Items for Systematic Reviews and Meta-analyses (PRISMA) flow diagram of the review process is presented in Fig. 1. Supplementary Table S1 presents the inclusion criteria based on the PICOS aspects (participants, interventions, comparators, outcomes, and study design) as recommended by the Cochrane Handbook for Systematic Reviews. The search strategy yielded 54 citations, of which 30 were excluded as it was clear from scrutinizing the title and abstract that they did not fulfill the selection criteria. Full manuscripts of 24 articles were obtained. A total of 19 of these publications were excluded because eight were reviews^5,10–16^, four articles reported findings on animal studies^17–20^, three articles were conference abstracts with limited data^21–23^, three articles did not specify outcomes from assisted reproductive techniques^24–26^, and one was a case-series report^27^. After exclusion of the inappropriate studies, five were included in the meta-analysis^4,6–9^.

The eligible studies were published between 2006 and 2017, and the number of patients included in the study ranged from 35 to 663 patients. Two studies were randomized controlled trials (RCTs)^7,8^, and three were nonrandomized cohort studies^4,6,9^. The randomization method was reported in one^7^ of two RCTs. Inclusion criteria based on the number of previous failed IVF cycles and the primary outcome varied among studies. None of the studies performed a power analysis. Financial support was declared in one^4^ out of five studies. The risk of bias assessments for RCTs and observational studies are summarized in Supplementary Tables S2 and S3.

The data extracted from each included study are listed in Tables 1 and 2.

**Table 1:** Study characteristics and summary of results.

Study, Country of origin	Study period	No of patients	Indication	Study design	Mean age (years)*	Timing of IU PBMC administration	Embryo transfer	No embryos transferred	Summary of results
Yashioka *et al*. (Japan) 2006	NA	35	≥4 previous failures, FSH < 15 mIU/ml	Cohort	37.5 vs. 36.6	3 days before ET	Fresh on Day 5	1–3	CPR and LBR were significantly higher in the PBMC group compared with controls (41.2% vs. 11.1% and 35.3% vs. 5.5%, respectively). Miscarriage rate was not reported.
Okitsu *et al*. (Japan) 2011	May 2007-Feb 2010	253	≥1 previous failures, FSH < 15 mIU/ml	Cohort	35.7 vs. 35.7	2 days before ET	Frozen-thawed on Day 3 or 5	1-2	CPR and LBR were not different for both groups (34.9% vs. 32.9% and 21.7% vs. 21.8%, respectively). However, for women with ≥3 RIF, PBMCs were associated with a significant increase in CPR (42.1% vs. 25%), although the difference was not significant for LBR (21.2% vs. 11.1%). Miscarriage rate was not reported.
Madkour *et al*. (Morocco) 2015	NA	54	≥2 previous failures, <40 y	RCT	42.5 vs. 41.6	2 days before ET	Fresh on Day 3	1–3	CPR was significantly higher in the PBMC group compared with controls (44.4% vs. 14.8%). The benefit is stronger for women with ≥3 RIF (70% vs. 29%). Miscarriage risk was lower in the PBMC group (17% vs. 75%). LBR was not reported.
Yu *et al*. (China) 2016	Sep 2013-May 2014	240	≥3 previous failures, <35 y, FSH < 15 mIU/ml	RCT	31.2 vs. 31.1	1 day before ET	Frozen-thawed on Day 3	1–3	CPR and LBR were significantly higher in the PBMC group compared with controls (46.2% vs. 20.9% and 34.4% vs. 14.3%, respectively). Miscarriage rate was not different between the two groups (20.9% vs. 31.8%).
Li *et al*. (China) 2017	July 2013-Mar 2015	663	≥1 previous failures, FSH < 15 mIU/ml	Cohort	30.8 vs. 30.5	1 day before ET	Frozen-thawed on Day 3 or 5	1–3	CPR was significantly higher in the PBMC group compared with controls (47.6% vs. 39.2%), but LBR was similar (33.0% vs. 31.6%). In women with ≥4 RIF, PBMCs were associated with significant increases in CPR (39.6% vs. 14.3%) and LBR (33.3% vs. 9.6%). Miscarriage rate was not reported.

NA: Not available, FSH: Follicle-stimulating hormone, RCT: Randomized controlled trial, IU: Intrauterine, PBMCs: Peripheral blood mononuclear cells, ET: Embryo transfer, CPR: Clinical pregnancy rate, LBR: Live birth rate, RIF: Recurrent implantation failure, *Mean age for intervention vs. control group: no significant difference was reported, **Body mass index was not reported in any study.

**Table 2:** Methodological differences in studies.

	Ovarian stimulation	Oocyte maturation trigger	Fertilization	hCG culture (hours)	IU PBMC administration	Mean PBMNC concentration (x10^6^)	Timing of IU PBMC administration	Embryo freezing
Yoshioka *et al*.^4^	GnRH agonists	5000 IU hCG	IVF/ICSI	48	2 × 10^7^ cells in 200 µl	10	3 days before ET	None
Okitsu *et al*.^6^	Both GnRH agonists and antagonists	5000 IU hCG	IVF/ICSI	None	3 × 10^7^ cells in 500 µl	6	2 days before ET	Vitrification/slow freezing
Madkour *et al*.^7^	GnRH antagonist	10,000 IU hCG	ICSI	72	1 × 10^6^ cells in 400 µl	0.25	2 days before ET	None
Yu *et al*.^8^	NA	NA	NA	24	1–2 × 10^7^ cells in 200 µl	7.5	1 day before ET	NA
Li *et al*.^9^	Both GnRH agonists and antagonists	5000 IU hCG	IVF/ICSI	24	1–2 × 10^7^ cells in 200 µl	7.5	1 day before ET	Vitrification

GnRH: Gonadotropin-releasing hormone, NA: Not available, hCG: Human chorionic gonadotropin, IVF: *In vitro* fertilization, ICSI: Intracytoplasmic sperm injection, IU: Intrauterine, PBMCs: Peripheral blood mononuclear cells, ET: Embryo transfer.

#### Live birth rate

**Figure 2:**
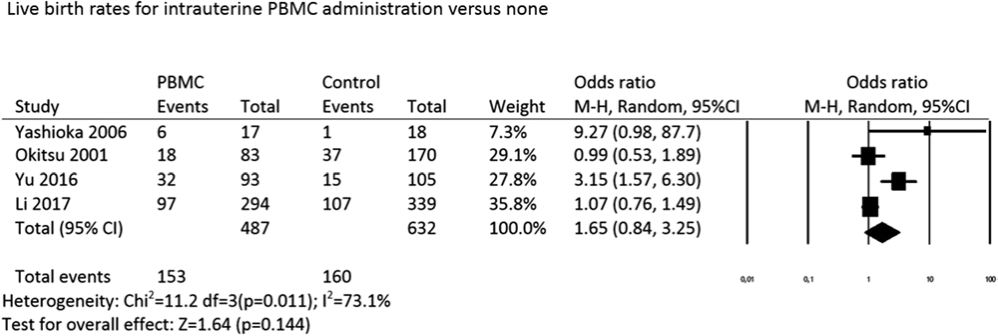
Live birth rates for intrauterine PBMC administration versus none.

The meta-analysis regarding the LBR is derived from four studies: one RCT^8^ and three nonrandomized cohort studies^6,9^ (Fig. 2). No difference in LBRs was noted between PBMC-treated and control groups (OR: 1.65, 95% CI: 0.84–3.25; p = 0.14; random effects model; heterogeneity; *I*^2^: 73.1%).

#### Live birth rates in frozen-thawed embryo transfer cycles

**Figure 3:**
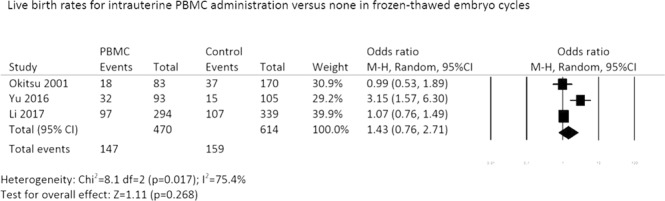
Forest plot of live birth rates for intrauterine PBMC administration versus none in frozen-thawed embryo cycles.

When the analysis was restricted to frozen-thawed embryo transfer cycles, LBRs were similar between PBMC-treated and control groups (OR: 1.43, 95% CI: 0.76–2.71; P = 0.26; random effects model; heterogeneity; *I*^2^: 75.4%) (Fig. 3).

#### Clinical pregnancy rate

**Figure 4:**
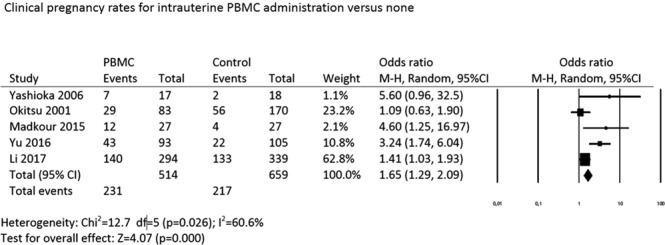
Clinical pregnancy rates for intrauterine PBMC administration versus none.

All five studies were included in the meta-analysis for clinical pregnancy rate (CPR) outcome (Fig. 4). Pooling the data together, a significant improvement in the clinical pregnancy rate in women who had been given intrauterine PBMCs before embryo transfer was noted compared with those who had not (OR: 1.65, 95% CI: 1.30–2.10; p = 0.001, heterogeneity; *I*^2^: 60.6%).

#### Clinical pregnancy rate in women with ≥3 failures

**Figure 5:**
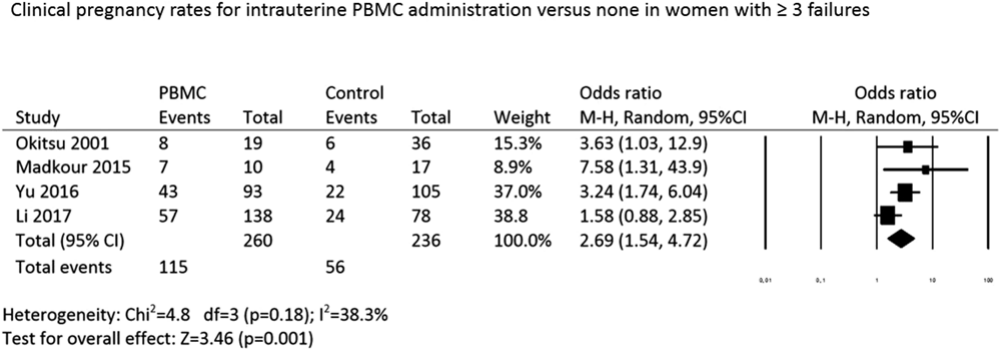
Forest plot for clinical pregnancy rates for intrauterine PBMC administration versus none in women with ≥3 failures.

Regarding the clinical pregnancy rate in the subgroup of patients with ≥3 failures, meta-analysis of the four studies, including two RCTs^7,8^ and two observational studies^6,9^, demonstrated a significant benefit of intrauterine PBMC administration (OR: 2.69, 95% CI: 1.53–4.72; P = 0.001; random effects model; heterogeneity; *I*^2^: 38.3%) (Fig. 5).

#### Miscarriage rate

**Figure 6:**
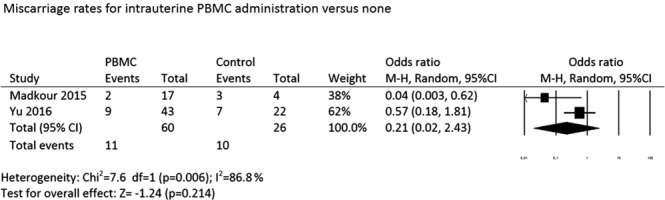
Miscarriage rates for intrauterine PBMC administration versus none.

Pooled estimates for the miscarriage risk revealed no significant difference between PBMC-treated and control groups (OR: 0.21, 95% CI: 0.02–2.43; P = 0.21; random effects model) (Fig. 6). However, the sample size was very low, and significant heterogeneity (*I*^2^ = 86.8%) was observed among studies.

None of the studies reported any adverse events.

## Discussion

The present systematic review and meta-analysis summarizes the best available evidence regarding the intrauterine administration of PBMCs in cases with RIF. The results from the included studies demonstrated that intrauterine administration of PBMCs with or without hCG culture fails to improve live birth rates when compared to controls. However, PBMCs were associated with an improvement in clinical pregnancy rates, which was more prominent in women with ≥3 failures.

Our analysis was strengthened by a number of factors. A comprehensive search strategy was used, employing relevant research databases. Additionally, a valid data synthesis method was implemented, and no language restrictions were applied. Cochrane’s and ROBINS-I tools were used to assess the quality of the included studies, suggesting a low to moderate risk of bias^28,29^.

There are also weaknesses in our analysis, which mainly arise from the high level of clinical heterogeneity of the publications that were included. There were many sources of heterogeneity, including the patient population (number of previous failed IVF cycles), study design (randomized or nonrandomized), intervention (*in vitro* culture with or without hCG, dose, volume and timing of PBMC administration, day and number of embryo transfers, fresh or frozen-thawed embryo transfer) and primary and secondary outcomes. The small sample size and substantial differences in the study design or patient populations may also undermine the credibility of the results. Another possible limitation of the review is that although multiple scientific databases were used in the electronic search, some relevant reports might still have been missed.

Several lines of evidence from both animal models and *in vitro* studies suggest improvements in implantation rates following intrauterine PBMC administration. Pregnancy rates and embryonic implantation sites were significantly increased after intrauterine PBMC treatment in bovine^17,18^ and mouse models^19^. PBMCs promote murine blastocyst spreading and invasion as well as human choriocarcinoma-derived BeWo cell invasion *in vitro*, and these promoting effects were augmented by hCG supplementation^20^. Various mechanisms have been proposed to explain the implantation-promoting effects of PBMCs. PBMC treatment induces the production of several cytokines, such as IL-1α, IL-1ß, and TNF- α, which can have positive impacts on endometrial receptivity and actively contribute to blastocyst attachment and invasion^5,18,19^. Further, hCG activates PBMC sin vitro and promotes trophoblast invasion through enhancement of LIF and IL-1 ß secretion^20,24^. PBMCs cultured with hCG might modulate the implantation site and infiltrate into endometrial stroma, serving as a guide for the invading blastocyst. In addition, regulatory Th-2 cells, which are PBMC-derived immune cells, may facilitate a more permissive immune-inflammatory profile for implantation^25,26^. Although the biological plausibility of modulating the immune response to implantation is intriguing, the magnitude of the clinical effect is yet to be determined.

The positive impact of intrauterine PBMC administration on pregnancy rates might be attributed to two other factors. The first factor is the mechanical trauma induced on the endometrium by intrauterine catheterization before embryo transfer. The proposed mechanism here is the modulation of the local immune system by a proinflammatory response to catheter-induced injury through secretion of cytokines, growth factors, interleukins and immune cells, such as macrophages and dendritic cells, which would favor implantation^30^. Madkour *et al*. suggested that this argument could be refuted based on the lack of a positive effect on implantation rates in women with one or two IVF failures, whereas a significant enhancement was observed with the same technique in women with ≥3 IVF failures^7^. A Cochrane analysis, which included nine RCTs on 1496 women, showed that endometrial injury performed between day 7 of the previous cycle and day 7 of the ET cycle was associated with an increase in live birth or ongoing pregnancy rates (RR 1.42, 95% CI 1.08 to 1.85) compared with no intervention or a sham procedure^31^. Another Cochrane review that meta-analyzed nine trials testing the efficacy of the method in subfertile women and couples attempting to conceive through sexual intercourse or intrauterine insemination reported that it is uncertain whether it improves the chance of a live birth or ongoing pregnancy^32^. The most recent data from a large, multicenter, well-designed, randomized controlled trial failed to show an improvement in the clinical outcome by endometrial scratching, reporting similar LBRs for both the intervention and control groups (26.1% (180/690) vs. 26.1% (176/674), respectively; odds ratio = 1.00 [0.78 to 1.27])^33^.

The second factor is the effect of hCG administered into the uterine cavity prior to embryo transfer. hCG, the key molecule to initiate the communication between the embryo and the endometrium, contributes to maternal tolerance of the embryo through interactions with immune cells within the receptive endometrium and plausibly in systemic circulation. A Cochrane review including 12 RCTs of 4038 women analyzed the efficacy of the intrauterine administration of hCG in ART. The study, which also suffered from a considerable degree of heterogeneity (*I*^2^ > 75%), reported an increase in clinical pregnancy (RR 1.41, 95% CI 1.25 to 1.58) and live birth rates (RR: 1.57, 95% CI 1.32 to 1.87) in the subgroup of women having cleavage-stage embryo transfers with intrauterine hCG administration compared with women with no hCG treatment^14^. However, they concluded that the current evidence for intrauterine hCG treatment does not support its use in ART cycles due to the small size and the variable quality of the trials and the fact that positive findings were extracted from a subgroup analysis. The other systematic review and meta-analysis on the subject analyzed eight RCTs that included 3087 women and found no difference in the live birth (RR 1.13; 95% CI 0.84 to 1.53) and spontaneous abortion rates (RR 1.00, 95% CI 0.74 to 1.34) in women who received intrauterine hCG and those who did not^15^.

Could these data be considered as new evidence of a game changer in patients with RIF? Based on the results of the present meta-analysis, there is limited evidence suggesting that intrauterine administration of PBMCs with or without hCG culture improves LBRs in women with RIF. However, the reliability of the data is limited by the observational nature of the available studies.

As infertile couples and their physicians have long been awaiting opportunities to celebrate medical breakthroughs, it is crucial for infertility specialists to refrain from offering treatment options that are not evidence based. The scientific background and the available evidence should be shared with the couple prior to implementing such a procedure. The use of PBMCs should be considered within the context of clinical trials with proper randomization and patient consent. Furthermore, there is a lack of long-term safety data of PBMC administration that is needed.

## Methods

### Databases and search strategies

The searches and selection of the studies were performed independently by two of the reviewers (K.Y. and O.O.), and any disagreement was resolved by discussion. PubMed, Web of Science and Cochrane library databases (from inception to April 2017) were searched for all relevant articles under the following key words and/or medical subject heading (MeSH) terms: ‘assisted reproduction’ or ‘IVF’ or ‘intracytoplasmic sperm injection’ or ‘embryo transfer’, or ‘implantation failure’ or recurrent implantation failure’ or ‘intrauterine administration’ and ‘peripheral blood mononuclear cells’ or ‘peripheral mononuclear cells’. References of all included primary and review articles were examined to identify relevant articles not captured by the electronic search. In addition, reference lists of all relevant publications and review articles as well as meeting proceedings of the European Society of Human Reproduction and Embryology and the American Society for Reproductive Medicine were hand-searched for the identification of relevant studies. No restriction was applied in terms of language, geographic distribution or publication type.

This study was performed using a predetermined protocol in accordance with the PRISMA reporting guidelines (Supplementary Table S4).

### Inclusion and exclusion

The study was designed a priori with inclusion of primary articles that studied women undergoing any form of ART (IVF, ICSI) who had intrauterine administration of PBMCs with or without hCG culture before fresh or frozen embryo transfer. Adjuvant medical therapy should not have been administered and asymmetric intervention should not have been performed in the control group. Eligible study designs were randomized controlled trials (RCTs), nonrandomized controlled trials, and prospective controlled cohort studies. The inclusion criteria based on the PICOS aspects are summarized in Supplementary Table S1.

### Data extraction and quality assessment

Two review authors (K.Y. and O.O.) independently extracted the following data from the included studies: demographic data (citation data, country, study period, number of patients included), study design, methodology (method of randomization, allocation concealment), intervention (administration route, timing, amount, extraction, *in vitro* culture, culture conditions), ovarian stimulation protocol, type and starting dose of gonadotropin administered for ovarian stimulation, type and dose of medication used for triggering final oocyte maturation, criteria used for triggering, type of fertilization, endometrial preparation protocol, day of embryo transfer, embryo quality, embryo freezing and thawing techniques and protocols, type of luteal support, adverse events associated with the intervention and pregnancy outcome.

The main outcome measure chosen for the current meta-analysis was live births per patient. Secondary outcomes were the clinical pregnancy rate (evidence of an intrauterine pregnancy sac with fetal heart activity at 6–8 weeks of gestation) or miscarriage rate. In cases of missing information, the study authors were contacted to retrieve relevant data where available. Any disagreement between the two reviewers regarding data extraction was resolved by discussion.

To determine the validity of the included trials, we assessed the risk of bias by Cochrane’s tool for the ‘risk of bias’ assessment in the RCTs^10^ and the ROBINS-I tool in the observational studies^11^.

#### Statistical analysis

All outcomes were dichotomous, and the results were expressed for each trial as an odds ratio (OR) with a 95% confidence interval (CI). To examine the associations between intrauterine PBMC administration and outcomes of interest, the OR with 95% CI was estimated by summarizing the risk estimates of each study using the random effect model. Heterogeneity of exposure effects was evaluated graphically using forest plots^34^ and heterogeneity across studies using the *I*^2^ statistic. Five studies were included in the meta-analysis; therefore, a funnel plot asymmetry test was not performed^35^. To investigate the possible sources of heterogeneity, we performed stratified analyses for the following: fresh versus frozen embryo transfer and <3 or ≥3 previous treatment failures. Statistical analyses were performed using RevMan 5.1 software (Cochrane Collaboration, Oxford, UK).

## Supplementary information

Dataset 1
