# Supplementary material for: Intrauterine administration of peripheral mononuclear cells in recurrent implantation failure: a systematic review and meta-analysis
Source: Sci Rep. 2019 Mar 7;9:3897. doi: 10.1038/s41598-019-40521-w (PMC6405957; doi:10.1038/s41598-019-40521-w)
Supplement: Supplementary file 1 — Dataset 1 [file 41598_2019_40521_MOESM1_ESM.pdf]

Intrauterine administration of peripheral mononuclear cells in recurrent implantation failure: a systematic review and meta-analysis

Kayhan Yakin, Ozgur Oktem, Bulent Urman

Supplementary Table S1. Inclusion criteria based on the PICOS aspects

Study characteristics	Inclusion
Population	• Women with recurrent implantation failure undergoing any form of assisted reproductive treatment (ART) such as in vitro fertilization (IVF) or intracytoplasmic sperm injection (ICSI)
Intervention	• Intrauterine administration of peripheral blood mononuclear cells (PBMCs) with or without human chorionic gonadotropin (hCG) culture before fresh or frozen embryo transfer
Comparison	• No intervention<br>• No adjuvant medical therapy
Outcome	• Live birth rate (primary)<br>• Clinical pregnancy rate (secondary)<br>• Miscarriage rate (secondary)
Study design	• Clinical human studies<br>• Randomized controlled trials<br>• Nonrandomized trials<br>• Prospective controlled cohort studies<br>• No language restriction

Supplementary Table S2. Risk of bias assessment of the randomized controlled trials included in the systematic review and meta-analysis.

Study	Selection bias		Performance bias	Detection bias	Attrition bias	Reporting bias
	Random sequence generation	Allocation concealment	Blinding of participants and personnel	Blinding of outcome	Incomplete outcome data	Selective reporting
Madkour et al. (2015)	High	High	High	High	Low	Low
Yu et al. (2016)	Unclear	Unclear	High	High	Low	Low

Assessed using the Cochrane 'Risk of Bias' assessment tool (Higgins and Green, 2012).

Supplementary Table S3. The ROBINS-I tool for assessing the risk of bias of observational studies included in the systematic review and meta-analysis.

Study ID	Study type	Pre-intervention		At intervention	Post-intervention				Total score
		Confounding bias	Selection bias	Classification bias	Deviation bias	Missing data bias	Measurement of outcome bias	Selective reporting bias	Overall risk of bias judgment
Yashioka et al. (2006)	Cohort	Moderate risk	Moderate risk	Low risk	Low risk	Moderate risk	Moderate risk	Moderate risk	Moderate risk[a]
Okitsu et al. (2011)	Cohort	Low risk	Moderate risk	Low risk	Low risk	Moderate risk	Moderate risk	Low risk	Moderate risk[a]
Li et al. (2017)	Cohort	Moderate risk	Moderate risk	Low risk	Low risk	Moderate risk	Moderate risk	Serious risk	Serious risk[b]

[a]Moderate risk: the study is judged to be at low or moderate risk of bias for all domains.
[b]Serious risk: the study is judged to be at serious risk of bias in at least one domain but not at critical risk of bias in any domain.

Supplementary Table S4. PRISMA 2009 Checklist*

Section/topic	#	Checklist item	Reported on page #
**TITLE**			
Title	1	Identify the report as a systematic review, meta-analysis, or both.	Page 1, the title defines the study as a systematic review and meta-analysis.
**ABSTRACT**			
Structured summary	2	Provide a structured summary including, as applicable: background; objectives; data sources; study eligibility criteria, participants, and interventions; study appraisal and synthesis methods; results; limitations; conclusions and implications of key findings; systematic review registration number.	Page 1, first paragraph provides a structured summary of the study. Systematic review registration number is not available.
**INTRODUCTION**			
Rationale	3	Describe the rationale for the review in the context of what is already known.	Page 1, paragraphs 2-3;<br>Page 2, paragraph 1 provides the rationale for the review and scientific background
Objectives	4	Provide an explicit statement of questions being addressed with reference to participants, interventions, comparisons, outcomes, and study design (PICOS).	Page 2, paragraph 2 defines the objective of the study
**METHODS**			
Protocol and registration	5	Indicate if a review protocol exists, if and where it can be accessed (e.g., Web address), and, if available, provide registration information including registration number.	Page 5, paragraph 5 and page 6, paragraphs 1-2 define the review

			protocol. Systematic review registration number is not available.
Eligibility criteria	6	Specify study characteristics (e.g., PICOS, length of follow-up) and report characteristics (e.g., years considered, language, publication status) used as criteria for eligibility, giving rationale.	Page 6, paragraph 3 defines the eligibility criteria. Detailed criteria based on PICOS format are presented in Supplementary Table S1
Information sources	7	Describe all information sources (e.g., databases with dates of coverage, contact with study authors to identify additional studies) in the search and date last searched.	Page 5, paragraph 5 and page 6, paragraph 1 describe information sources
Search	8	Present full electronic search strategy for at least one database, including any limits used, such that it could be repeated.	Page 5, paragraph 5 and page 6, paragraphs 1-2 describe the search strategy
Study selection	9	State the process for selecting studies (i.e., screening, eligibility, included in systematic review, and, if applicable, included in the meta-analysis).	Page 6, paragraph 3; Figure 1; and Supplementary Table 1 describe study selection and eligibility criteria
Data collection process	10	Describe method of data extraction from reports (e.g., piloted forms, independently, in duplicate) and any processes for obtaining and confirming data from investigators.	Page 6, paragraphs 5-6; subheading data extraction and quality assessment
Data items	11	List and define all variables for which data were sought (e.g., PICOS, funding sources) and any assumptions and simplifications made.	Page 6, paragraphs 5-6; subheading data extraction and quality assessment
Risk of bias in individual studies	12	Describe the methods used for assessing the risk of bias of individual studies (including specification of whether this was done at the study or outcome level), and how this information is to be used in any data synthesis.	Page 7, paragraph 1; Supplementary Table S2; and Table S3 describe the risk of bias assessment
Summary measures	13	State the principal summary measures (e.g., risk ratio, difference in means).	Page 7, paragraph 2 describes the principal summary measures

Synthesis of results	14	Describe the methods of handling data and combining the results of studies, if done, including measures of consistency (e.g., I^2) for each meta-analysis.	Page 7, paragraph 2 describes the analytic measures
Risk of bias across studies	15	Specify any assessment of the risk of bias that may affect the cumulative evidence (e.g., publication bias, selective reporting within studies).	Page 7, paragraph 1; Supplementary Table S2; and Table S3 describe the risk of bias assessment
Additional analyses	16	Describe the methods of additional analyses (e.g., sensitivity or subgroup analyses, meta-regression); if done, indicate which were pre-specified.	-
**RESULTS**			
Study selection	17	Give numbers of studies screened, assessed for eligibility, and included in the review, with reasons for exclusions at each stage, ideally with a flow diagram.	Page 2, paragraph 4 describes study selection, and Figure 1 presents the flow diagram
Study characteristics	18	For each study, present characteristics for which data were extracted (e.g., study size, PICOS, follow-up period) and provide the citations.	Page 2, paragraph 5 and Table 1 define study characteristics
Risk of bias within studies	19	Present data on risk of bias of each study; if available, any outcome level assessment (see item 12).	Supplementary Table S2 and Table S3 summarize risk of bias assessment of each study
Results of individual studies	20	For all outcomes considered (benefits or harms), present for each study: (a) simple summary data for each intervention group and (b) effect estimates and confidence intervals, ideally with a forest plot.	Tables 1 and 2 present and compare the study characteristics and outcomes.
Synthesis of results	21	Present results of each meta-analysis done, including confidence intervals and measures of consistency.	Page 3, Figures 2-6 present a synthesis of the results
Risk of bias across studies	22	Present results of any assessment of risk of bias across studies (see Item 15).	Supplementary Table S2 and Table S3

Additional analysis	23	Provide results of additional analyses, if done (e.g., sensitivity or subgroup analyses, meta-regression [see Item 16]).	-
**DISCUSSION**			
Summary of evidence	24	Summarize the main findings including the strength of evidence for each main outcome; consider their relevance to key groups (e.g., healthcare providers, users, and policy makers).	Page 3, the first paragraph of Discussion
Limitations	25	Discuss limitations of study and outcome level (e.g., risk of bias), and at review-level (e.g., incomplete retrieval of identified research, reporting bias).	Page 4, paragraphs 1-2
Conclusions	26	Provide a general interpretation of the results in the context of other evidence, and implications for future research.	Page 4, paragraphs 3-4<br>Page 5 paragraphs 1-4
**FUNDING**			
Funding	27	Describe sources of funding for the systematic review and other support (e.g., supply of data); role of funders for the systematic review.	Page 9

*Moher, D. Liberati, A., Tetzlaff, J., Altman, D.G., The PRISMA Group. Preferred Reporting Items for Systematic Reviews and Meta-Analyses: The PRISMA Statement. *BMJ* 339:b2535 (2009).
